# Intravenous iron for acute and chronic heart failure with reduced ejection fraction (HFrEF) patients with iron deficiency: An updated systematic review and meta-analysis

**DOI:** 10.1016/j.clinme.2024.100211

**Published:** 2024-04-21

**Authors:** Ahmed K. Awad, Mahmoud Shaban Abdelgalil, Ahmed R. Gonnah, Adel Mouffokes, Unaiza Ahmad, Ayman K. Awad, Merihan A. Elbadawy, David Hesketh Roberts

**Affiliations:** a Faculty of Medicine, Ain-Shams University, Cairo, Egypt; b Imperial College Healthcare NHS Trust, London, United Kingdom; c Faculty of Medicine, University of Oran 1 Ahmed Ben Bella, Oran, Algeria; d Punjab Medical college, Faisalabad, Pakistan; e Faculty of Medicine, El-Galala University, Suez, Egypt; f Lancashire Cardiac Centre, Blackpool, United Kingdom; g University of Liverpool, Liverpool, United Kingdom

**Keywords:** Intravenous iron, Iron deficiency, Acute, Chronic, HFrEF

## Abstract

Patients with heart failure (HF) and iron deficiency are at increased risk of adverse clinical outcomes. We searched databases for randomised controlled trials that compared IV iron to placebo, in patients with HF with reduced ejection fraction (HFrEF). A total of 7,813 participants, all having HFrEF with 3,998 receiving IV iron therapy, and 3,815 control recipients were included. There was a significant improvement in Kansas City Cardiomyopathy Questionnaire favouring IV iron with MD 7.39, 95% CI [3.55, 11.22], *p* = 0.0002. Subgroup analysis, based on acute and chronic HF, has displayed a sustained statistical significance. Additionally, a significant increase in the left ventricular ejection fraction % was observed, with MD 3.76, 95% CI [2.32, 5.21], *p* < 0.00001. A significant improvement in 6-min walk test was noted, with MD 34.87, 95% CI [20.02, 49.72], *p <* 0.00001. Furthermore, IV iron showed significant improvement in NYHA class, peak VO_2_, serum ferritin, and haemoglobin levels. Finally, despite the lack of difference in terms of all-cause hospitalisation and HF-related death, IV iron was associated with a significant reduction in HF-related, any cardiovascular reason hospitalisations, and all-cause death; which supports the need for implementation of IV iron as a standard of care in patients with HF and iron deficiency.

## 1 Introduction

Iron deficiency (ID) is the most common micronutrient deficiency worldwide, affecting approximately one-third of the global population.^1^ Iron deficiency (ID) is acknowledged as an important comorbidity in patients with heart failure (HF), regardless of their ejection fraction.^2^, ^3^, ^4^, ^5^, ^6^ Furthermore, patients with HF, and concomitant iron deficiency are at an increased risk for hospitalisation, and mortality in both acute and chronic heart failure.^7^^,^^8^ IDA has also been found to have a negative impact on the clinical heart failure status, adversely affecting the patients’ quality of life.^9^, ^10^, ^11^ This has now become a global health concern, associated with undesirable consequences, both physically and socially.^12^ IDA in HF can be absolute, due to depletion of iron storage, or functional due to insufficient iron uptake into erythrocytes; despite adequate total body iron levels.^13^^,^^14^ Absolute and functional IDA in HF causes can be attributed to numerous aetiologies, although the underlying mechanisms are not fully understood.^15^

For many years, oral iron supplementation in ferrous form has been the mainstay treatment in the management of IDA. Two trials - the IRON-HF and the IRONOUT-HF trials^16^^,^^17^ - have been conducted, to assess the safety and efficacy of oral iron in patients with HF, and concomitant iron deficiency. The findings of these trials have shown oral iron to be ineffective in patients with HF.^17^ Newer formulations of intravenous (IV) iron have evolved using mainly ferric carboxymaltose (FCM), with a number of trials showing improved symptoms and with faster regression of iron deficiency. Therefore, we conducted our meta-analysis to evaluate the safety, and the efficacy of IV FCM on clinical endpoints in heart failure patients.

## 2 Methods

The Preferred Reporting Items for Systematic Reviews and Meta-Analyses (PRISMA) guidelines were followed, in this systematic review and meta-analysis (Table S1).^18^ Moreover, we prospectively published our protocol on OSF (DOI: doi.org/10.17605/OSF.IO/9K2D3).

### 2.1 Data sources, search strategy

We searched PubMed, the Cochrane Library, Scopus, and EMBASE for randomised controlled trials (RCTs) that compared the administration of IV iron to placebo; in patients with acute and chronic heart failure with reduced ejection fraction (HFrEF). The search was conducted from inception to 13 December 2023. The following search terms were used: ('intravenous iron' OR 'IV iron') AND ('Heart failure' OR 'HF' OR 'reduced Ejection fraction HF').

### 2.2 Eligibility criteria

Studies that investigated the efficacy of IV iron versus placebo in patients with heart failure, and had enough data for qualitative and quantitative analysis were included. No language restrictions were used. Our PICOS (population, intervention, comparison, outcome, and study type) criteria were: P: patients with heart failure; I: IV iron with its different types; C: Placebo; O: Primary outcomes were the mean change from baseline in Kansas City Cardiomyopathy Questionnaire (KCCQ), New York Heart Association class (NYHA class), serum ferritin, serum transferrin saturation, Left ventricular ejection fraction (LVEF) %, 6-min walk test (6-MWT), haemoglobin, and peak oxygen consumption (VO_2_). Secondary outcomes included all-cause hospital admission, death, and death due to worsening HF, hospitalisation for any cardiovascular reason; S: Randomised controlled trials (RCTs). Comparative observational studies, unpublished articles, letters to the editor, reviews, animal studies, and meta-analyses were excluded.

### 2.3 Study selection

Five authors (A.K.A, A.R.G, A.K.A, M.A.E, and M.S.A) independently screened the titles, and abstracts of the retrieved records, followed by a full-text screening of the potentially eligible records. Any conflicts were resolved by discussion.

### 2.4 Data extraction

Five authors (A.M, A.K.A, A.R.G, M.A.E, and A.K.A) extracted the following data from the included studies: summary of included studies and baseline characteristics: name of the first author, publication year, country, study design, age, sex, LVEF, haemoglobin, 6-min walk test distance, serum ferritin saturation, estimated glomerular filtration rate, and NYHA class. Furthermore, for qualitative and quantitative analysis, we extracted post treatment variables to calculate the mean change from baseline for our primary outcomes. We extracted the rates of all-cause hospital admission, death, death due to worsening HF, hospitalisation for any cardiovascular reason; as well as any disorders for our secondary outcomes with any discrepancy in the results being resolved through discussion.

### 2.5 Risk of bias and quality assessment

To assess the quality of observational studies, we used the Newcastle-Ottawa Scale (NOS).^19^ The studies were evaluated by star allocation on three perspectives, with nine being the highest possible quality: (1) selection of study groups (0–4 points), (2) comparability of cohorts (0–2 points), and (3) establishment of the outcome of interest (0–3 points). Observational studies having four stars for selection, two stars for comparability, and three stars for the outcome were considered to have a low risk of bias. Studies with two or three stars for selection, one star for comparability, and two stars for outcome, were considered to have a medium risk of bias. Finally, studies with a score of one for selection, outcome ascertainment, or zero for any of the three domains were considered to have a high risk of bias.

For the RCTs, we used the revised Cochrane Risk of Bias Tool for RCTs (RoB 2.0).^20^ RoB 2.0 addresses five domains: (1) bias arising from the randomisation process; (2) bias due to deviation from the intended interventions; (3) bias due to missing the outcome data; (4) bias in the measurement of the outcome; and (5) bias in the selection of the reported result. Three independent reviewers (A.K.A, A.R.G, and A.M) assessed the methodological quality of included studies, and any discrepancies were resolved through discussion.

### 2.6 Statistical analysis

The meta-analysis was conducted using RevMan (version 5.4).^21^ We pooled dichotomous outcomes, using odds ratio (OR), and continuous outcomes using mean difference (MD), which were presented with the corresponding 95% confidence interval (CI). We used the I-square and chi-square tests to examine the heterogeneity. The Chi-square test determines if there is substantial heterogeneity, while the I-square determines the magnitude of heterogeneity. A substantial heterogeneity (for the Chi-square test) is defined as an alpha level below 0.1, according to the Cochrane Handbook (chapter nine).^22^ The I-square test is interpreted as follows: (0–40%: not significant; 30–60%: moderate heterogeneity; 50–90%: significant heterogeneity). We utilised the fixed-effects model due to low statistical heterogeneity in all of our outcomes. We also conducted a subgroup analysis stratifying the included studies based on nature of heart failure, either acute or chronic, however, only patients with reduced ejection fraction were included.

## 3 Results

### 3.1 Literature search results

After conducting an electronic search on multiple databases, we obtained a total of 937 articles. Out of these, 42 duplicates were eliminated, resulting in 895 articles, that were title and abstarct screened for relevance. 870 irrelevant articles were excluded based on the title and abstract screening. 25 articles required a full-text screening, and 18 RCTs were included in both the qualitative, and quantitative analysis, as shown in Supplementary Fig. 1.

### 3.2 Summary of the included studies

18 RCTs studies^23^, ^24^, ^25^, ^26^, ^27^, ^28^, ^29^, ^30^, ^31^, ^32^, ^33^, ^34^, ^35^, ^36^, ^37^, ^38^, ^39^, ^40^ yielded a total of 7,813 participants, all having HFrEF with 3,998 receiving IV iron therapy, and 3,815 control recipients. The mean age of the population included was 68.2 years. The baseline characteristics of patients included can be found in Table 1, along with the summary of the included studies in Supplementary Table 2.

**Table 1:** Baseline characteristics of enrolled patients in each included studies.

Author name, year	Treatment arms	Number of patients n (%)	Age, mean (SD)	Males *n* (%)	Type of heart failure	Ejection fraction, %, mean (SD)	Haemoglobin, g/L, mean (SD)	6 min walk test distanc, mean (SD)	Serum ferritin, mg/L, mean (SD)	Transferrin saturation, %, mean (SD)	Estimated glomerular filtration rate, mL/mi*n*, mean (SD)	NYHA class, *n*(%)		
												II	III	IV
**Kalra 2022**	IV iron	569	73.2 (66.7–80.1)*	427 (75)	Acute	32 (25–37)*	12.1 (11.2–12.8)	NA	49.0 (30.0–86.0)	15(11–20)*	51.7 (38.1–68.1)*	328 (58)	230 (40)	11 (2)
	Placebo	568	73.5 (67.1–79.1)*	410 (72)		35(26–38)*	12.1 (11.2–12.9)*	NA	50.0 (30.0–85.0)	15(10–19)*	50.1 (37.8–68.6)*	320 (56)	238 (42)	10 (2)
**Akintunde 2021**	IV iron	30	65.5 (19.1)	2	Acute	44.6(10.1	9.4(2.6	156.9(72.5	NA	NA	NA	NA	NA	NA
	Placebo	30	61.2 (16.1)	7		41.6(8.2	11.6(1.5	254.7(106.7	NA	NA	NA	NA	NA	NA
**Martens 2021**	IV iron	37	72 (12)	26 (70)	Acute	33 (8)	13.3 (1.2	NA	82 (38–106)*	18.8(6.0)	56 (25)	22 (59)	15 (41)	NA
	Placebo	38	73 (9)	25 (66)		34 (7)	13.1 (1.3	NA	81 (43–99)*	19.4(7.0)	51 (22)	19 (50)	19 (50)	NA
**Ponikowski 2020**	IV iron	558	71.2 (10.8)	314 (56)	Acute	32.6	12.3 (1.6)	NA	83.9 (62.2)	15.2(8.3)	NA	255 (46)	272 (49)	NA
	Placebo	550	70.9 (11.1)	300 (55)		32.7	12.1 (1.6)	NA	88.5 (68.6)	14.2(7.5)	NA	240 (44)	277 (50)	NA
**Yeo 2018**	IV iron	24	61.1 (10.8)	18 (75)	Acute	38.8 (17.5)	11.6 (1.9)	252.4 (122.7)	91.4 (80.4)	15.7(10.1)	NA	NA	NA	NA
	Placebo	25	64 (10)	20 (80)		33.2 (14.8)	13.1 (1.3)	242.6 (66.8)	84.1 (63.7)	13.9(6.8)	NA	NA	NA	NA
**Ponikowski 2015**	IV iron	150	68.8 (9.5)	83	Acute	37.1 (7.5)	12.37 (1.41)	288 (98)	57.0 (48.4)	20.2 (17.6)	66.4 (21.7)	54 (63)	70 (47)	NA
	Placebo	151	69.5 (9.3)	77		36.5 (7.3)	12.42 (1.30)	302 (97)	57.1 (41.6)	18.2 (8.1)	63.5 (20.9)	91 (60)	60 (40)	
**Beck-da-Silva 2013**	IV iron	17	65.511.8	(70.5)	Chronic	26.8(7.89)	11.24(0.54)	NA	150.4(143.668)	18.8(8.98)	NA	NA	NA	NA
	Placebo	6	68.9 (10.1)	(66.7)		30.7 (7.4)	10.9 (0.7)	NA	95 (128)	13.5 (5.8)	NA	NA	NA	NA
**Veldhuisen 2017**	IV iron	86	63(12)	60 (70)	Chronic	33(9)	12.9(1.3)	NA	48	17.3	52(13)	61 (71)	25 (29)	NA
	Placebo	86	64(11)	69 (80)		31(8)	13.0(1.5)	NA	53	18.1	51(12)	54 (63)	32 (37)	NA
**Anker 2009**	IV iron	304	67.8(10.3)	145(47.7)	Chronic	31.9(5.5)	119(13)	274(105)	52.5(54.5)	17.7(12.6)	63.8(21.2)	53 (17.4)	251 (82.6)	NA
	Placebo	155	67.4(11.1)	70(45.2)		33.0(6.1)	119(14)	269(109)	60.1(66.5	16.7(8.4)	64.8(25.3)	29 (18.7)	126 (81.3)	NA
**Okonko 2008**	IV iron	24	64 (14)	17 (71)	Chronic	30 (7)	12.6 (1.2)	NA	62(37)	20(8)	NA	13 (54)	11 (46)	NA
	Placebo	11	62 (11)	8 (73)		29 (6)	12.2(1)	NA	88 (62)	21 (9)	NA	6 (55)	5 (45)	NA
**Toblli 2007**	IV iron	20	76 (7)	NA	Chronic	30.8(1.7)	10.3(0.6)	192.3 (60.9)	73.0 (29.9)	20 (1)	39.8(10.1)	NA	NA	NA
	Placebo	20	74(8)	NA		10.3(0.6)	10.2(0.5)	190.7(56.1)	70.6 (21.4)	20(1)	37.7(10.2)	NA	NA	NA
**Toblli 2015**	IV iron	30	75.4(6	NA	Chronic	30.2(3.5)	10.1(0.8)	NA	70.6(24.9)	19.2(1.8)	39.5(8.4)	26 (74.3)	NA	NA
	Placebo	30	74.7(7)	NA		29.9(3.2)	10.1(0.6)	NA	68.4(18.3)	19.2(1.9)	37.8(8.3)	30 (85.7)	NA	NA
**Charles Edwards 2019**	IV iron	21	70(12)	NA	Chronic	37(8)	130(15)	324(79)	59(32.03)	21(8)	37.8(7.3)	NA	10 (53)	NA
	Placebo	19	62(13)	NA		37(8)	128(20)	313(67)	34(25.44)	18(10)	NA	NA	9 (43)	NA
**Dahoot 2020**	IV iron	35	51.0 (11.6)	NA	Acute	24.9 (5)	11 (1.4)	431 (64.79)	40.1 (27.2)	NA	NA	NA	9 (25.7%)	NA
	Placebo	35	54.8 (9.0)	NA		25.8 (5.5)	11.3 (0.9)	418 (46.9)	45.5 (35.1)	NA	NA	NA	5 (14.3%)	NA
**Silverberg 2001**	IV iron	16	75.3 (14.6)	NA	Chronic	30.8(12.6)	10.3(1.2)	NA	221.4(165.1)	NA	NA	NA	NA	NA
	Placebo	16	72.2 (9.9)	NA		28.4(7.6)	10.9(0.8)	NA	264.0(162.5)	NA	NA	NA	NA	NA
**Marcusohn 2022**	IV iron	18	70 (64.0 -76.2)*	12 (66.7)	Acute	30 (15 - 50)*	11.9 (10.8 - 12.5)*	196.0 (175.0 - 277.9)	92 (50 -165)	12.6 (9.5 -14.8)	NA	NA	NA	NA
	Placebo	16	74.5 (70.5 - 82.5)*	11 (68.8)		40 (28 - 58)*	11.4 (10.2 - 12.5)*	220.1 (168.0 - 286.8)*	110 (55 - 177)*	14.0 (10.1 - 16.7)	NA	NA	NA	NA
**Núñez 2023**	IV iron	27	73.5 (64- 77)*	21 (77.8)	Chronic	40 (33.5-45)*	13.1 (11.9-13.4)*	NA	73.0 (56.0-126.0)*	15.7 (12.0- 19.2)*	59.4 (50.0-71.3)*	24 (88.9)	NA	NA
	Placebo	26	71 (67- 79)*	19 (73.1)		37 (32-43)*	13.4 (12.7-14.6)*	NA	47.8 (23.0, 114.0)*	15.4 (9.6- 20.0)*	64.1 (48.9-79.3)*	26 (100)	NA	NA
**Mentz 2023**	IV iron	1532	68.6 (10.9)	1026 (66)	Chronic	30.8 (7.0)	12.6 (1.4)	273.9 (109.7)	56.0 (47.3)	23.9 (11.2)	NA	797 (52)	711 (46.6)	22 (1.4)
	Placebo	1533	68.6 (11.2)	1002 (65.4)		30.6 (7.3)	12.5(1.4)	274.7(109.4)	57.3(51.4)	23.0(10.3)	NA	820(53.5)	692(45.2)	19(1.2)

SD; standard deviation, N; number, NA; not available.

⁎ Data are expressed as median (IQR value).

### 3.3 Quality assessment

According to the ROB-2 quality assessment tool, 15 out of our 18 studies displayed a low risk of bias, while two were found to have moderate risk of bias due, to missing outcome data, and measurement of outcome. One study has displayed a high risk of bias, based on selection, and the reporting of results. Further details about the risk of bias asssessment can be found in Supplementary Fig. 2.

### 3.4 Primary outcomes, change from baseline in

#### 3.4.1 Kansas City Cardiomyopathy Questionnaire (KCCQ)

Analysis of KCCQ includes seven studies, with a total of 568 patients in the IV iron arm and 431 patients in the placebo arm, displayed a significant increase in KCCQ favouring IV iron with MD 7.39, 95% CI [3.55, 11.22], *p =* 0.0002. High heterogeneity was observed (*p <* 0.00001, I^2^=98%), which could not be resolved by sensitivity analysis.

We performed subgroup analysis to assess the impact of IV iron administration on KCCQ, in patients with acute and chronic heart failure. In the subgroup analysis based on acute heart failure, IV iron has shown a significant increase in KCCQ, compared with placebo (MD = 7.14, 95% CI [2.20, 12.09], *p =* 0.005). The results were heterogeneous with *p <* 0.00001, I^2^ = 92%.

**Fig. 1:**
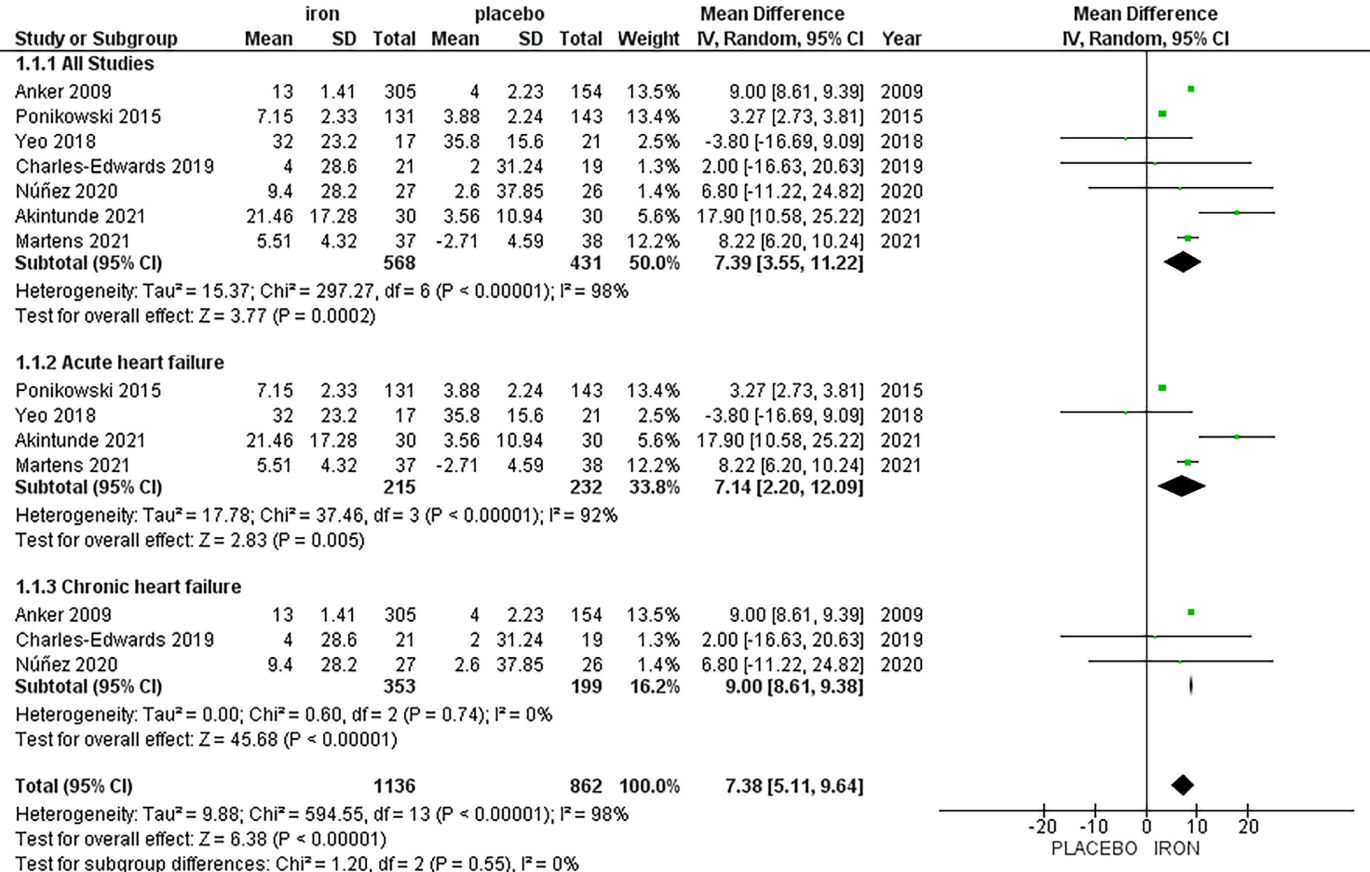
Forrest plots of analyses of Kansas City Cardiomyopathy Questionnaire (KCCQ).

In the subgroup analysis based on chronic heart failure, IV iron displayed a significant increase in KCCQ, compared with placebo (MD = 9.00, 95% CI [8.61, 9.38], *p <* 0.00001). The results were homogenous with *P* = 0.74, I^2^ = 0% (Fig. 1). Left ventricular ejection fraction (LVEF %).

**Fig. 2:**
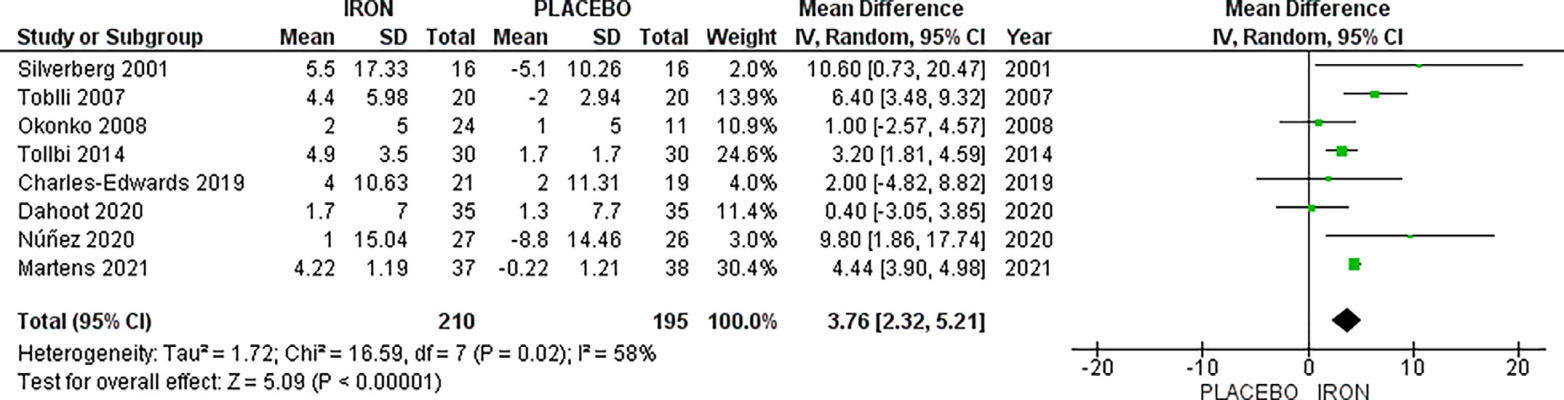
Left ventricular ejection fraction (LVEF%).

The analysis of LVEF % included eight studies, with a total of 210 patients in the IV iron arm and 195 patients in the placebo arm, displaying a significant increase in LVEF% favouring IV iron with MD 3.76, 95% CI [2.32, 5.21], *p <* 0.00001. High heterogeneity was observed (*p =* 0.02, I^2^ = 58%), which could not be resolved by sensitivity analysis (Fig. 2).

#### 3.4.2 6-min walk test (6MWT)

The analysis of 6MWT included nine studies, with a total of 2,111 patients in the IV iron arm and 1,951 patients in the placebo arm, displaying a significant increase in 6MWT favouring IV iron with MD 34.87, 95% CI [20.02, 49.72], *p <* 0.00001. High heterogeneity was observed (*p <* 0.00001, I^2^=97%), which could not be resolved by sensitivity analysis.

We performed subgroup analysis to assess the impact of IV iron administration on 6MWT in patients with acute and chronic heart failure. In the subgroup analysis based on acute heart failure, there was no significant difference between the two arms with MD = 94.75, 95% CI [−63.69, 253.18], *p =* 0.24. The results were heterogeneous with *p <* 0.00001, I^2^ = 97%.

**Fig. 3:**
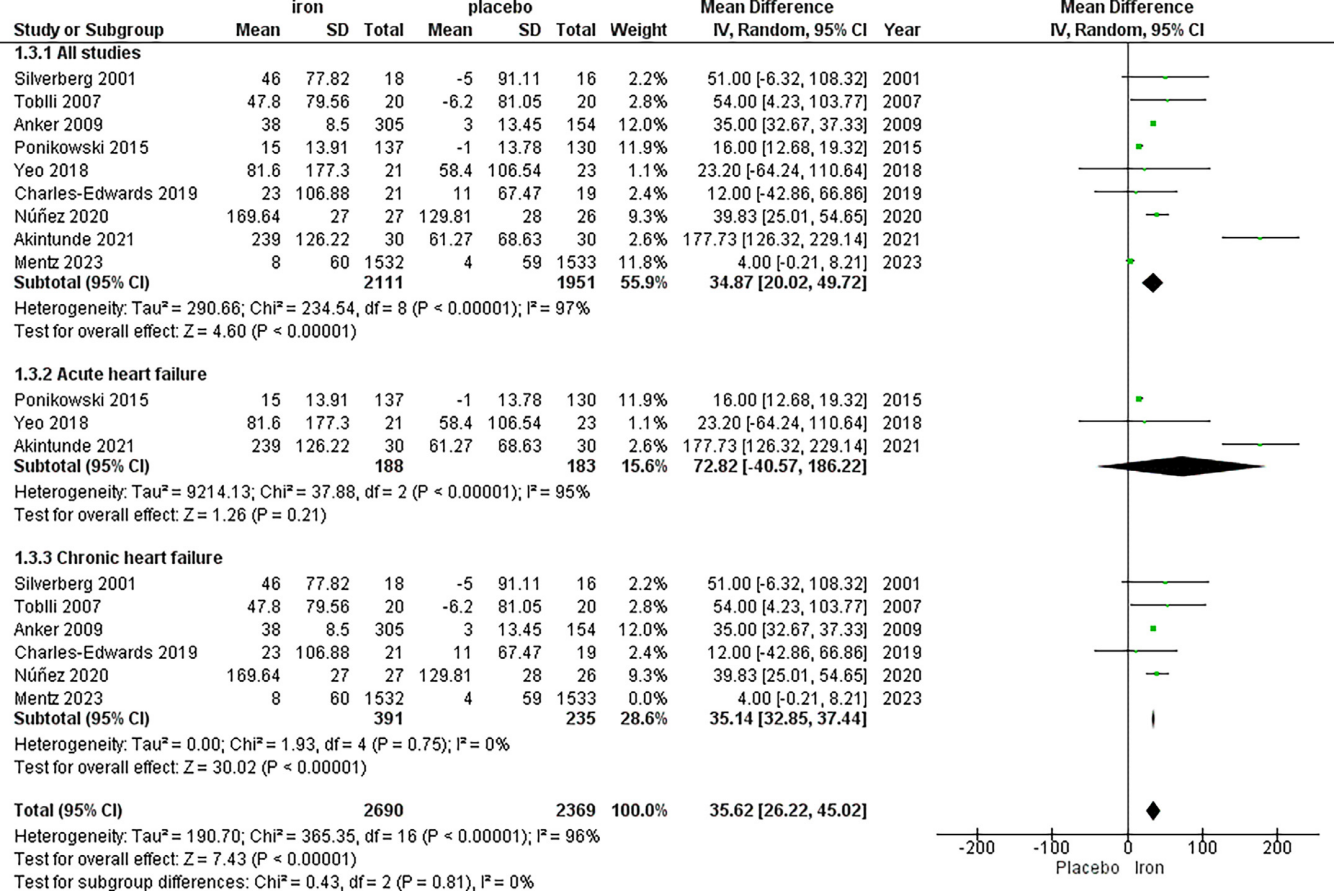
6-min walk test (6MWT).

In the subgroup analysis based on chronic heart failure, there was significant improvement for patients receiving IV iron with MD = 35.14, 95% CI [32.85, 37.44], *p <* 0.00001. The results were heterogeneous, yet it was solved to *p =* 0.75 and I^2^ = 0%, by removing Mentz *et al* 2023^40^ (Fig. 3).

#### 3.4.3 New York Heart Association class (NYHA class)

**Fig. 4:**
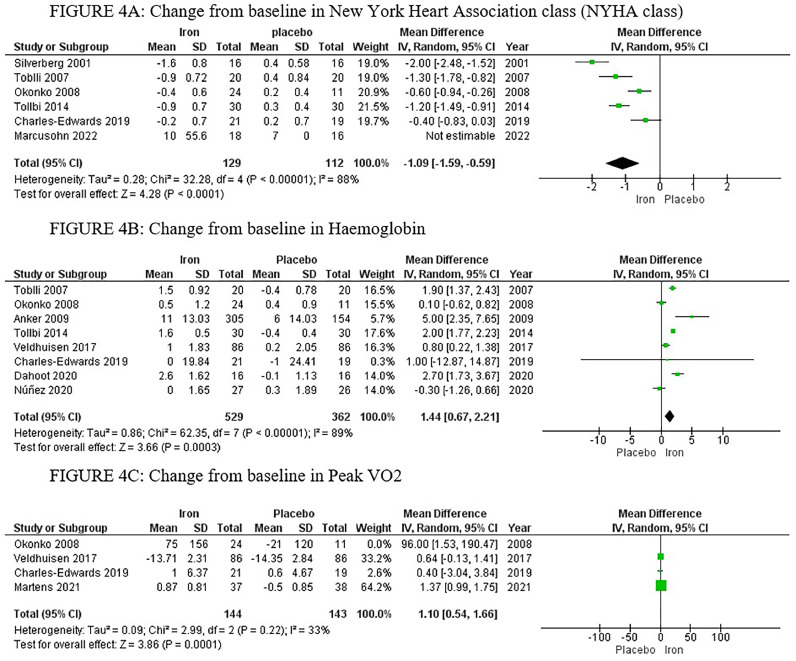
Forrest plots of analyses of A) New York Heart Association class (NYHA class) B) Haemoglobin C) Peak VO_2_.

The analysis of NYHA class included six studies, with a total of 129 patients in the IV iron arm and 112 patients in the placebo arm, displaying a significant improvement in NYHA class favouring the IV iron arm with MD −1.09, 95% CI [−1.59, −0.59], *p <* 0.00001. High heterogeneity was observed (*p <* 0.00001, I^2^=88%), which could not be resolved by sensitivity analysis (Fig. 4A).

#### 3.4.4 Haemoglobin levels

The analysis of haemoglobin levels included eight studies, with a total of 529 patients in the IV iron arm and 362 patients in the placebo arm, displaying a significant increase in haemoglobin levels, favouring the IV iron arm with MD 1.44, 95% CI [0.67, 2.21], *p =* 0.0003. High heterogeneity was observed (*p <* 0.00001, I^2^ = 89%), which could not be resolved by sensitivity analysis (Fig. 4B).

#### 3.4.5 Peak VO_2_

The analysis of peak VO_2_ included four studies, with a total of 168 patients in the IV iron arm, and 154 patients in placebo arm, displaying no significant difference between the two arms (MD 1.10, 95% CI [0.54, 1.66], *p =* 0.0001). High heterogeneity was observed (*p <* 0.00001, I^2^ = 89%), which was resolved by sensitivity analysis, by excluding Okonko *et al* 2008,^28^ with *p =* 0.22, I^2^=33% (Fig. 4C).

#### 3.4.6 Serum transferrin saturation

The analysis of serum transferrin saturation included seven studies, with a total of 267 patients in the IV iron arm and 448 patients in the placebo arm, displaying no significant difference between the two arms with MD −0.65, 95% CI [−5.75, 4.40], *p =* 0.79. High heterogeneity was observed (*p <* 0.00001, I^2^=97%), which could not be resolved by sensitivity analysis.

**Fig. 5:**
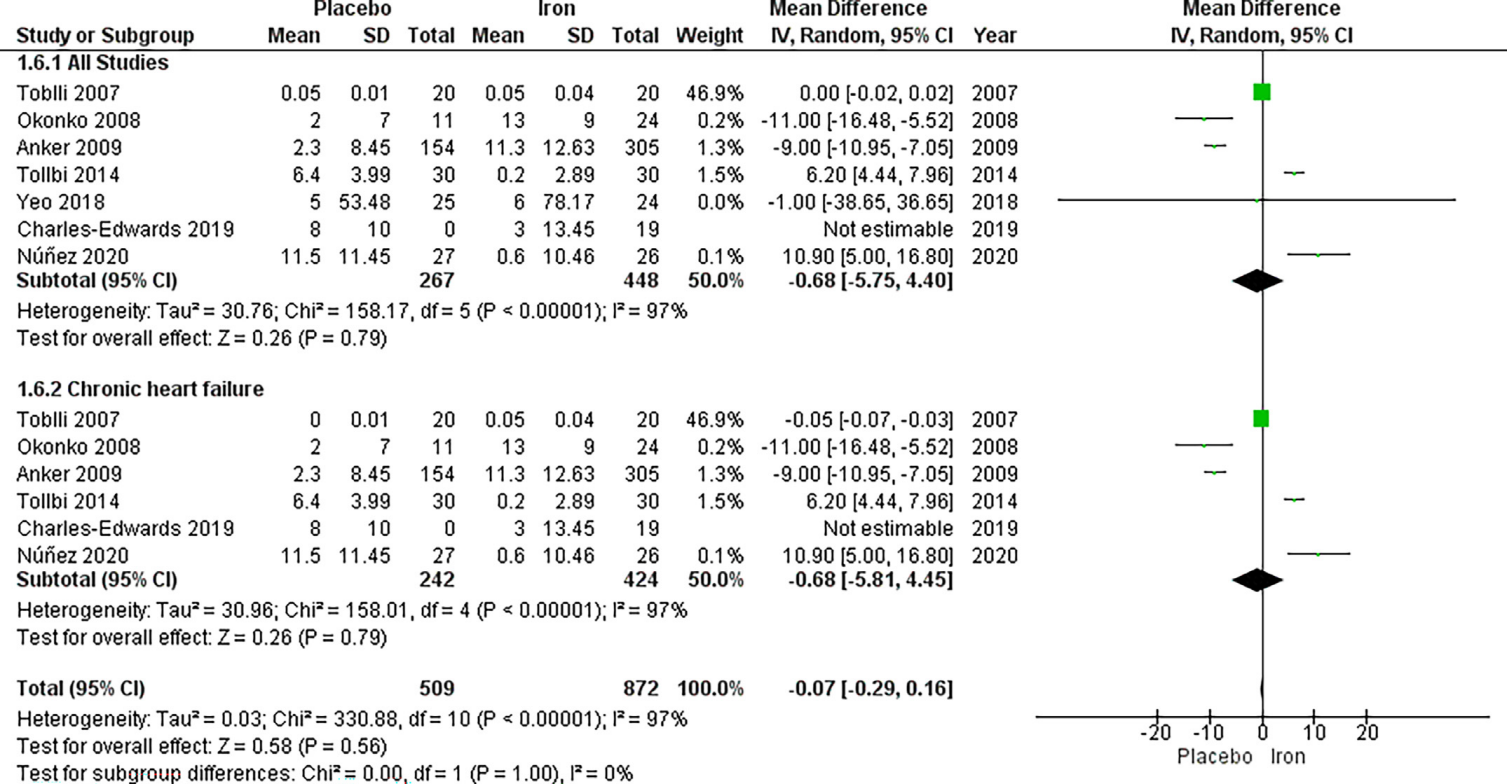
Forrest plots of analyses of serum transferrin saturation.

In the subgroup analysis based on chronic heart failure, no significant difference in serum transferrin was observed between IV iron and placebo (MD = −0.68, 95% CI [−5.81, 4.45], *p =* 0.79). The results were heterogeneous with *p <* 0.00001, I^2^ = 97% (Fig. 5).

#### 3.4.7 Serum ferritin

The analysis of serum ferritin included ten studies, with a total of 652 patients in the IV iron arm and 487 patients in the placebo arm, displaying a significant difference between the two arms favouring IV iron with (MD 152.65, 95% CI [107.67, 197.62], *p <* 0.00001). High heterogeneity was observed (*p <* 0.00001, I^2^ = 97%), which could not be resolved by sensitivity analysis.

**Fig. 6:**
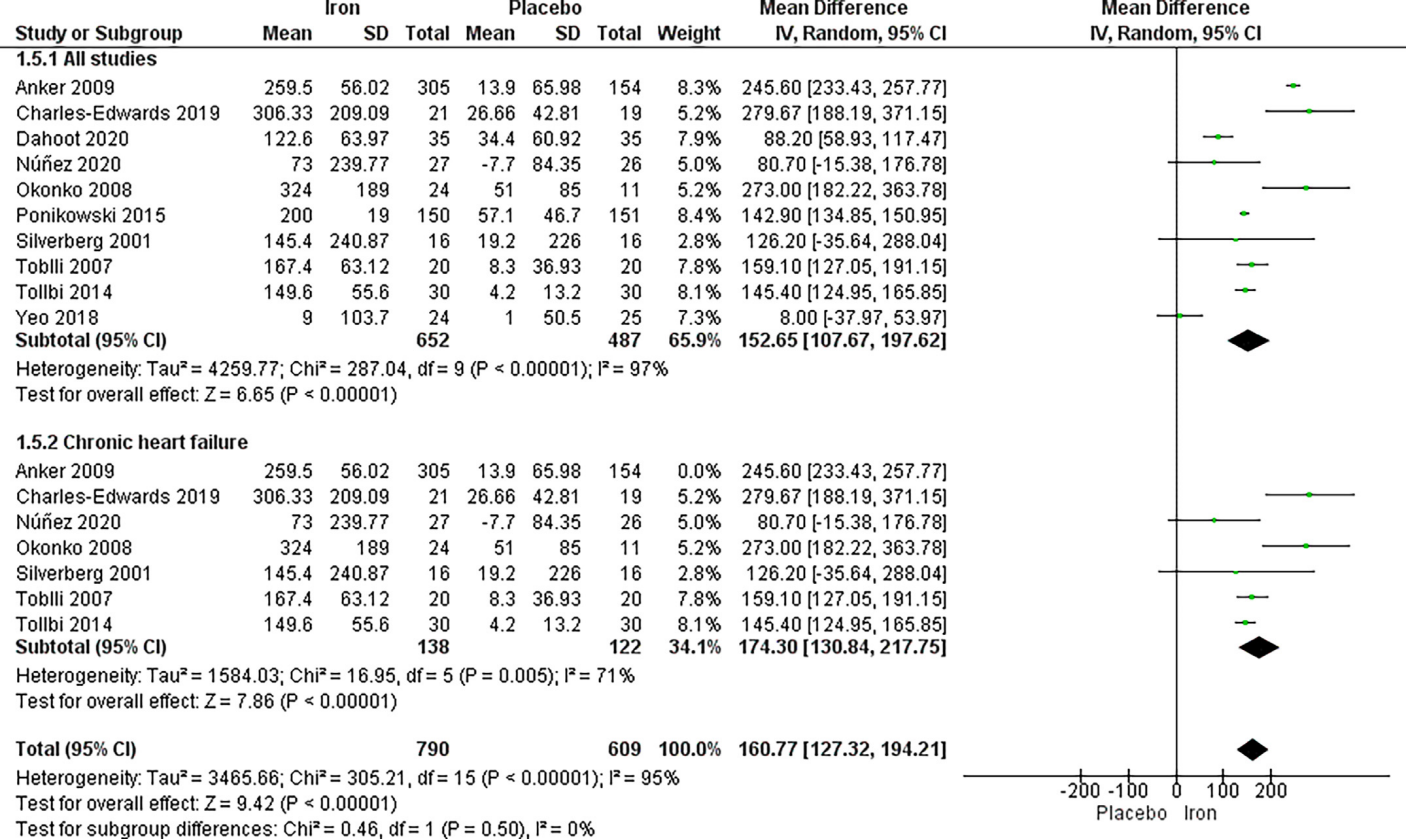
Serum ferritin.

In the subgroup analysis based on chronic heart failure, significant difference in serum ferritin was observed favouring IV iron (MD = 174.30, 95% CI [130.84, 217.75], *p <* 0.00001). The results were heterogeneous, which was resolved by removing Anker 2009,^38^ with *p =* 0.005, I^2^ = 71% (Fig. 6).

### 3.5 Secondary outcomes

#### 3.5.1 All-cause hospital admission

The analysis of all-cause hospital admission included four studies with a total of 980 patients in the IV iron arm and 828 patients in the placebo arm, displaying no significant difference between the two arms with RR 0.96, 95% CI [0.60, 1.53], *p =* 0.86. High heterogeneity was observed (*p =* 0.005, I^2^=76%), which could not be resolved by sensitivity analysis.

**Fig. 7:**
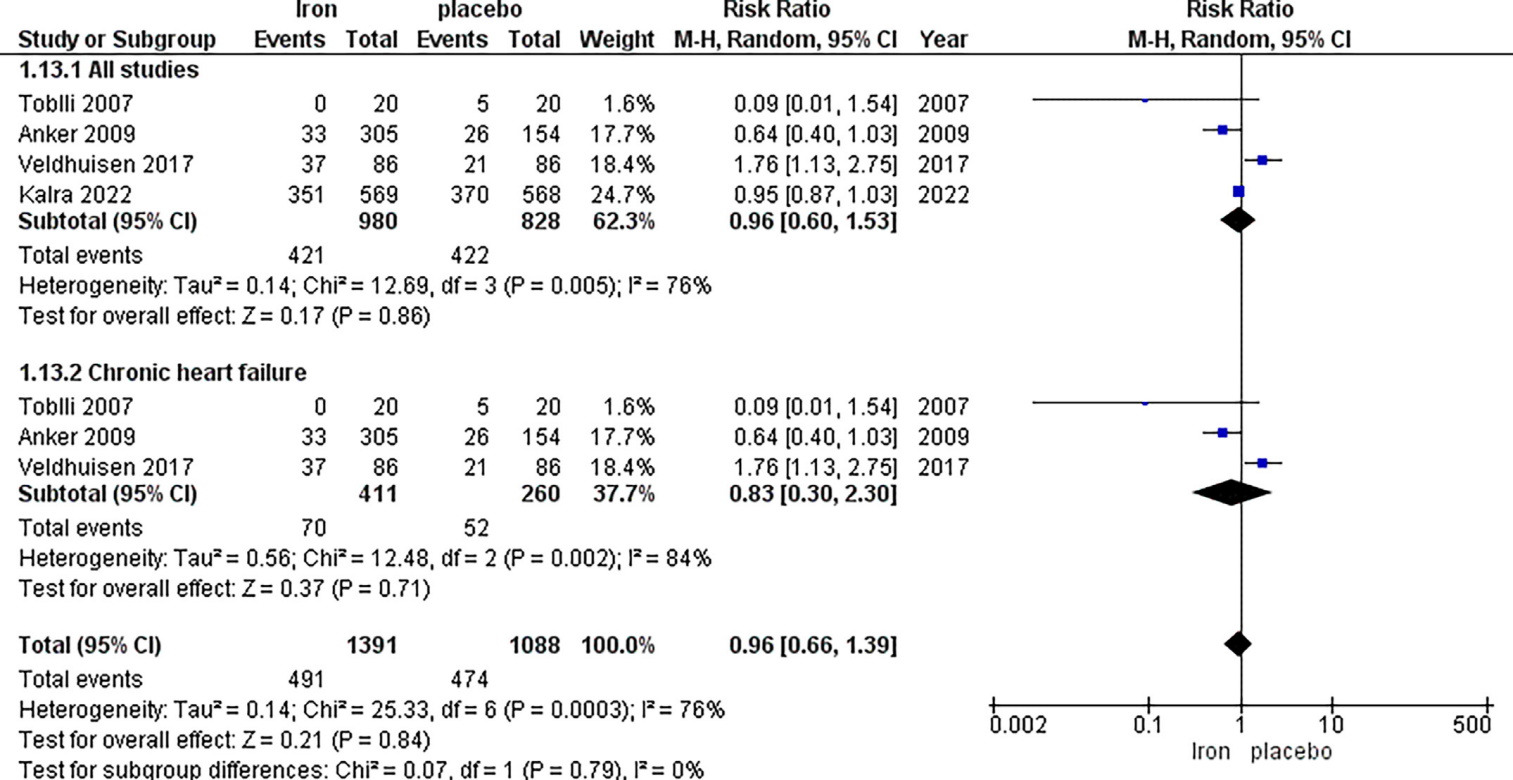
All-cause hospital admission.

In the subgroup analysis based on chronic heart failure, no significant difference in all-cause hospital admission was observed between IV iron and placebo (RR = 0.83, 95% CI [0.30, 2.30], *p =* 0.71). The results were heterogeneous with *p =* 0.002, I^2^ = 84% (Fig. 7).

#### 3.5.2 Hospitalisation for any cardiovascular reason

**Fig. 8:**
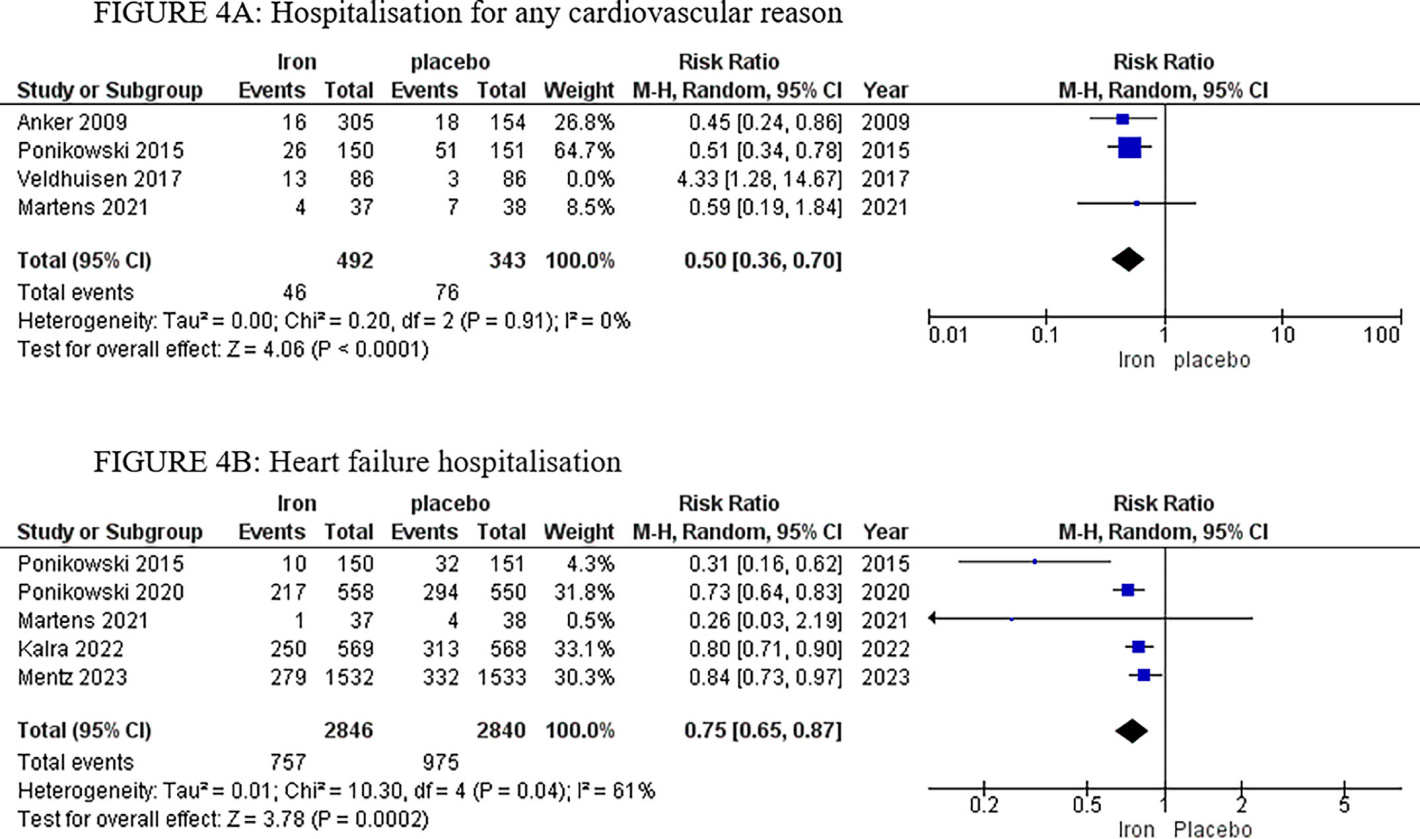
Forrest plots of analyses of A) Hospitalisation for any cardiovascular reason B) Heart failure hospitalisation.

The analysis of hospitalisation for any cardiovascular reason included four studies, with a total of 492 patients in the IV iron arm and 343 patients in the placebo arm, displaying a significantly lower risk in the IV iron arm with RR 0.50, 95% CI [0.36, 0.70], *p <* 0.0001. High heterogeneity was observed (*p =* 0.009, I^2^ = 74%), which was resolved by sensitivity analysis, by excluding Veldhuisen *et al* 2017,^31^ with *p =* 0.91, I^2^ = 0% (Fig. 8A).

#### 3.5.3 Heart failure hospitalisation

The analysis of heart failure hospitalisation included five studies, with a total of 2,846 patients in the IV iron arm and 2,840 patients in the placebo arm, displaying a significantly lower risk, favouring the IV iron arm with RR 0.75, 95% CI [0.65, 0.87], *p =* 0.0002. High heterogeneity was observed (*p =* 0.04, I^2^ = 61%) (Fig. 8B).

#### 3.5.4 Mortality

The analysis of mortality included eight studies, with a total of 2,174 patients in the IV iron arm and 2,014 patients in the placebo arm, displaying a significantly lower mortality risk, favouring IV iron (RR 0.80, 95% CI [0.65, 0.99], *p =* 0.04). No heterogeneity was observed (*p =* 0.42, I^2^ = 0%).

In the subgroup analysis based on acute heart failure, no significant difference in the mortality incidence was observed between IV iron and placebo (RR = 0.63, 95% CI [0.16, 2.42], *p =* 0.50). The results were homogeneous (*p =* 0.24, I^2^ = 27%).

**Fig. 9:**
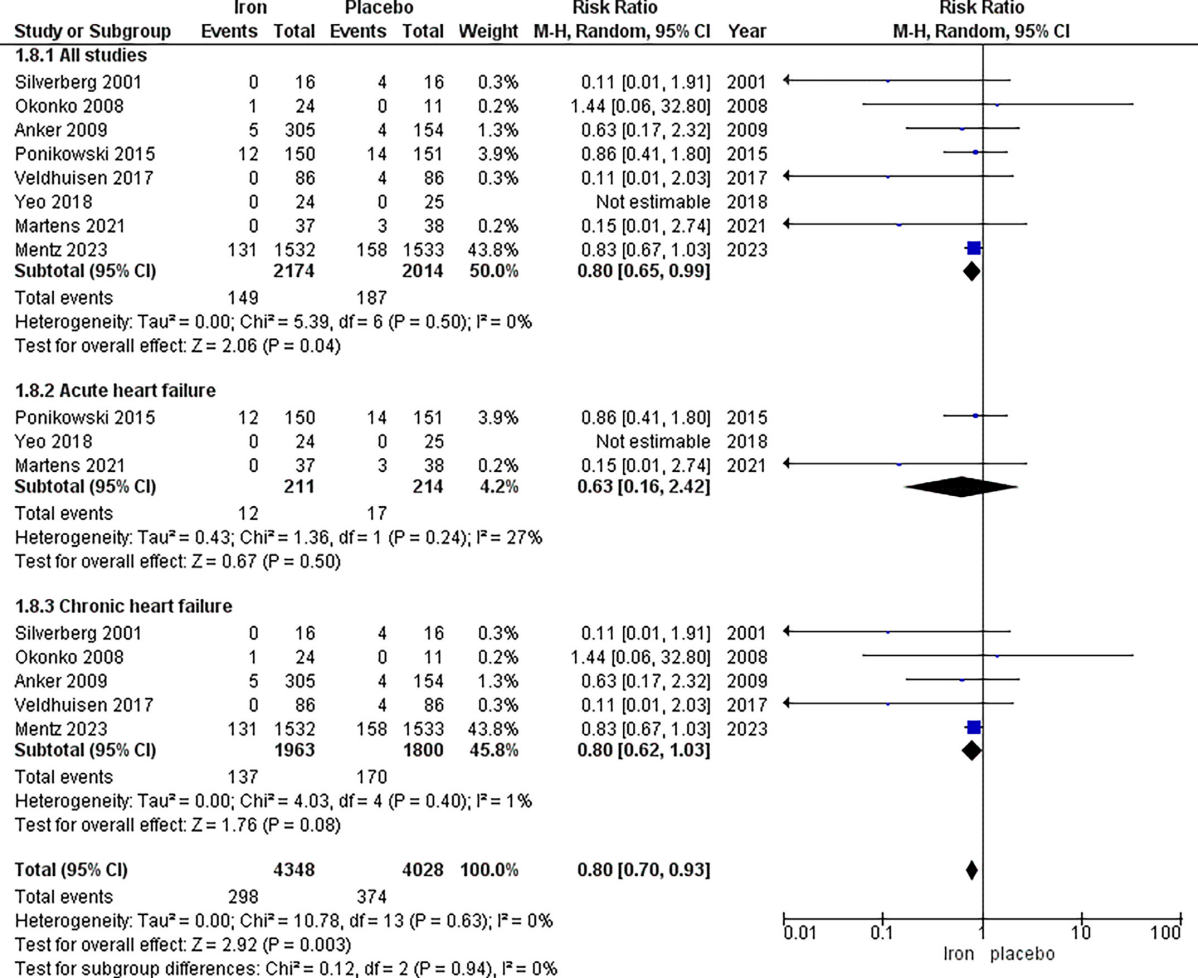
Forrest plots of analysis of mortality.

Additionally, in the subgroup analysis based on chronic heart failure, no significant difference in the mortality incidence was observed between IV iron and placebo (RR = 0.80, 95% CI [0.62, 1.03], *p =* 0.08). The results were homogeneous (*p =* 0.40, I^2^ = 1%) (Fig. 9).

#### 3.5.5 Mortality due to worsening HF

**Fig. 10:**
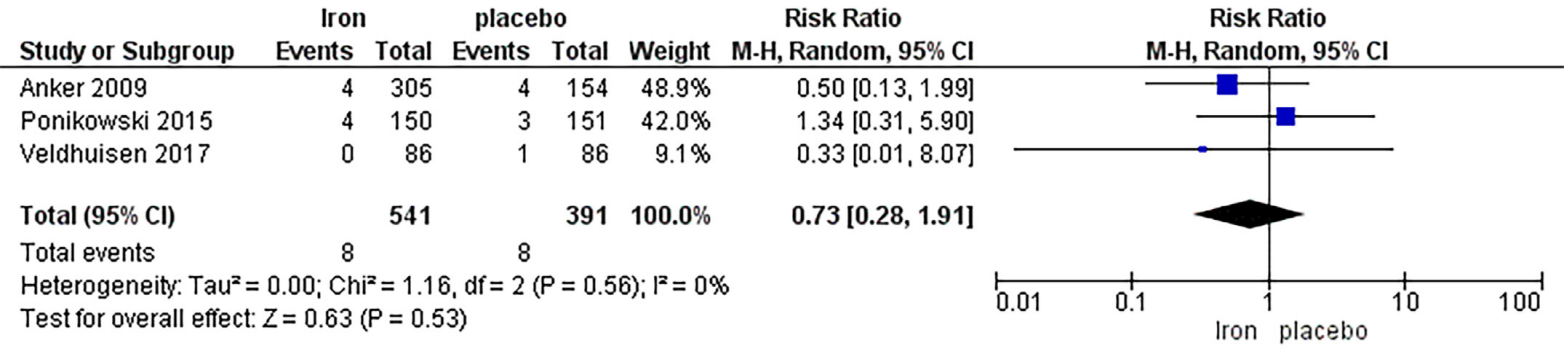
Forrest plot of analysis of mortality due to worsening heart failure.

The analysis of mortality due to worsening HF included three studies, with a total of 541 patients in the IV iron arm and 391 patients in the placebo arm, displaying no significant difference between the two arms with RR 0.73, 95% CI [0.28, 1.91], *p =* 0.53. Low heterogeneity was observed (*p =* 0.56, I^2^ = 0%) (Fig. 10).

## 4 Discussion

Iron deficiency affects up to 59% of patients with HF, and is independently associated with reduced exercise capacity, recurrent HF and any-cardiovascular reason hospitalisations, as well as increased cardiovascular and all-cause mortality.^41^ Our review including a total of 18 studies is the most up-to-date analysis of the efficacy of intravenous iron therapy in patients with HF. In a pooled analysis of 18 studies, we found a statistically significant improvement in the cardiac function of HF patients, which was assessed by an increase in LVEF%. However, despite the statistically significant improvement in LVEF %, which was ∼3%, the clinical significance (ie reduction in clinical HF symptoms, improved health-related quality of life (HRQoL), and functional status outcomes) remains debatable, given the reproducibility of echo, the main modality used to assess LVEF.

Additionally, patients' exercise endurance was significantly enhanced with the administration of IV iron therapy. Functional status parameters, such as NYHA class, demonstrated a significant improvement in the IV iron supplementation group. This was demonstrated by and significant improvement in peak consumption of oxygen VO_2_ values, 6MWT, and NYHA class which highlights improved mobility, as well as a marked reduction in HF symptoms; and hence a notable reduction in physical limitations. The effect on IV iron on 6MWT was notable only in the patients with chronic HF; however, there was a trend for improvement in the patients with acute HF, which needs to be further investigated in larger-scale trials in the future.

Moreover, in the IV iron group, the patients’ self-reported improvement in their health, which was shown by a statistically significant improvement in the KCCQ score, a heart-failure specific health-related quality of life tool. This statistical significance was maintained for both patients with acute and chronic heart failure, as displayed by our subgroup analysis. This reflects the improvement in mobility, ability to self-care, and the reduction in physical and social limitations which are clinically relevant in patients with HF, in conjuction with improvement in clinical outcomes.^11^ IV iron has significant increased serum ferritin and haemoglobin levels; however, other serum markers such as serum transferrin saturation did not differ statistically between the IV iron arm and placebo. Despite the lack of difference in all-cause hospitalisation and HF-related deaths, there was a significant reduction in the incidence of hospitalisation due to heart failure and any other cardiovascular reason with IV iron therapy. These are major outcomes which supports the need for implementation of use of IV iron in patients with HF and iron deficiency to improve patients’ outcomes, and to also reduce the burden on the healthcare services.

Our updated meta-analysis with a larger number of studies included provides confirmatory, as well as novel findings compared to previous studies in the literature.^41^, ^42^, ^43^, ^44^, ^45^ Previous meta-analyses^41^, ^42^, ^43^, ^44^, ^45^ have shown that (IV or oral) iron therapy did not influence mortality rates. There was also consistency of outcomes with previous studies^41^, ^42^, ^43^, ^44^, ^45^ in terms of improved hospitalisation rates, cardiac function (assessed by LVEF%), QoL and functional status further reinforcing the use of IV iron therapy in patients’ with reduced ejection fraction heart failure (LVEF<45%). However, our analysis has displayed a significant reduction in all-cause deaths which is a hard clinical outcome, further reinforcing the implementation of IV therapy as a standard of care worldwide in patients with HF, as most patients with HF have concomitant anaemia of chronic disease.^41^ Additonally, in contrast to Sindone *et al*, our study did not report a significant elevation in serum markers, such as percentage TSAT in IV iron treatment group, despite highlighting a consistent significance in terms of serum ferritin levels.^45^

The above mentioned improvements in cardiac status, and Qol can be attributed to its role in enhancement of cardiac contractility, and initiating cardiac reverse remodelling.^46^ Iron is an important component of cellular energetic mechanism being a key cofactor for enzymes involved in oxidative phosphorylation.^46^^,^^47^ Hence, iron deficiency decreases the ability of myocardium to contract especially during physical exertion.^46^ Data from animal models with ID suggest the inability for the heart to compensate with increased ATP, and phosphocreatine to meet the increased demands during exercise. This mismatch in heart failure with reduced ejection fraction results in low cardiac output, a phenomenon that research termed ‘negative force-frequency relationship’.^45^^,^^46^ Therefore, administration of IV iron improves cardiac contractility by correcting deficits on a molecular and cellular level. Martens *et al*^46^ and Lacour *et al*^47^ have reported reduced rate of cardiac reverse remodelling as recorded by a fall in LVEF, in HFrEF patients with concomitant ID after cardiac resynchronisation therapy (CRT). A plausible hypothesis of how intravenous administration of FCM counteracts it is hinted in an MRI study of patients with HFrEF, who demonstrated an improvement in LVEF subsequent to myocardial iron repletion visualised by T2-weighed MRI scans.^47^^,^^48^

The use of IV iron has displayed a significant benefit in terms of hospitalisation due to HF, and other cardiac causes as shown by our study, and other analyses.^41^, ^42^, ^43^, ^44^, ^45^ This benefit can reflect the impact of IV iron therapy on the quality of life of patients, as well as the healthcare services. ESC 2023 guidelines recommend using intravenous iron supplementation in HF patients with reduced, or moderately reduced ejection fraction, and iron deficiency, defined as serum ferritin <100 ng/mL or serum ferritin 100–299 ng/mL with TSAT <20%, to alleviate HF symptoms, improve exercise capacity and QoL, as well as reduce hospitalisation.^23^^,^^24^^,^^49^^,^^50^ However, this definition which is based on the FAIR-HF trial, in its current state is not without pitfalls due to its reliance on serum ferritin levels.^40^ Accurate identification of truly iron-deficient patients is a prerequisite for treatment, if positive outcomes are to be achieved. Additionally, IV iron should not be limited to only symptomatic patients, as iron can improve the outcomes if provided prophylactically in patients with HF. Furthermore, iron therapy has also shown benefit when used in patients with heart failure with preserved ejection fraction (significant improvement in diastolic function, exercise capacity and enhanced endothelial function due to reduced production of oxygen radical species).^51^ This warrants revision to the current guidelines.

Evidence from recent observational studies suggest that serum iron concentration, and TSAT might both be more strongly associated with prognosis than serum ferritin, and might be a better guide to which patients benefit from IV iron. This is because serum ferritin is affected by degrees of inflammation which is prevalent in patients with heart failure, and this can mask the clinical need of iron supplementation.^48^, ^49^, ^50^^,^^52^^,^^53^ As a result, higher cut-off values have been implemented for the definition of iron deficiency in patients with HF.^46^^,^^54^, ^55^, ^56^, ^57^ Another marker reflecting depleted intracellular iron can be high serum soluble transferrin receptors, and is increased in case of iron deficiency, rather than inflammation. High serum transferrin receptors identify patients at high risk of death beyond standard prognostic variables.^54^, ^55^, ^56^, ^57^ In addition, his analysis found that TSAT <20% and serum iron ≤13 µmol/L were associated with a higher mortality, and this was independent of HF phenotype, and this is when iron supplementation should be warranted to reduce the chance of mortality.^45^ This has important implications for future studies, to identify serum markers that can detect true iron deficiency accurately in clinical settings to prevent unintentional adverse effects.

## 5 Future studies

Despite the availability of various IV iron-carbohydrate formulations (FCM, Iron Isomaltoside, Iron sucrose etc.), there is a heavy preponderance of investigators towards FCM which is also recommended by the ESC guidelines.^49^ We propose that a comparative trial comparing the efficacy and safety of these three iron-carbohydrate therapies, with the additional identification of optimal doses should be conducted to guide better treatment strategies for physicians dealing with ID and HF. Moreover, the long-term impact of IV iron therapy should be further investigated, as the previous trials have not addressed this so far.

## 6 Limitations

Although we included only randomised controlled trials, baseline differences were observed especially in the type of heart failure either acute or chronic which we solved through sub-grouping each type separately. Moreover, the high heterogeneity in some of our analyses which we attempted to resolved through sensitivity analysis, by removing each study at a once to determine the study affecting the total effect estimate the most. The sensitivity analyses were unable to resolve the high heterogeneity in some instances, which is a limitation of our meta-analysis. Furthermore, all our heart failure patients are with reduced ejection fraction, thus the literature requires further RCTs to assess the efficacy of IV iron on patients with preserved ejection fraction as well. Lastly, we were unable to perform an individual patient data meta-analysis due to limited availability of data. We believe an individual participant data (IPD) meta-analysis will provide more nuance to guide therapies according to the patients.

## 7 Conclusion

**Fig. 11:**
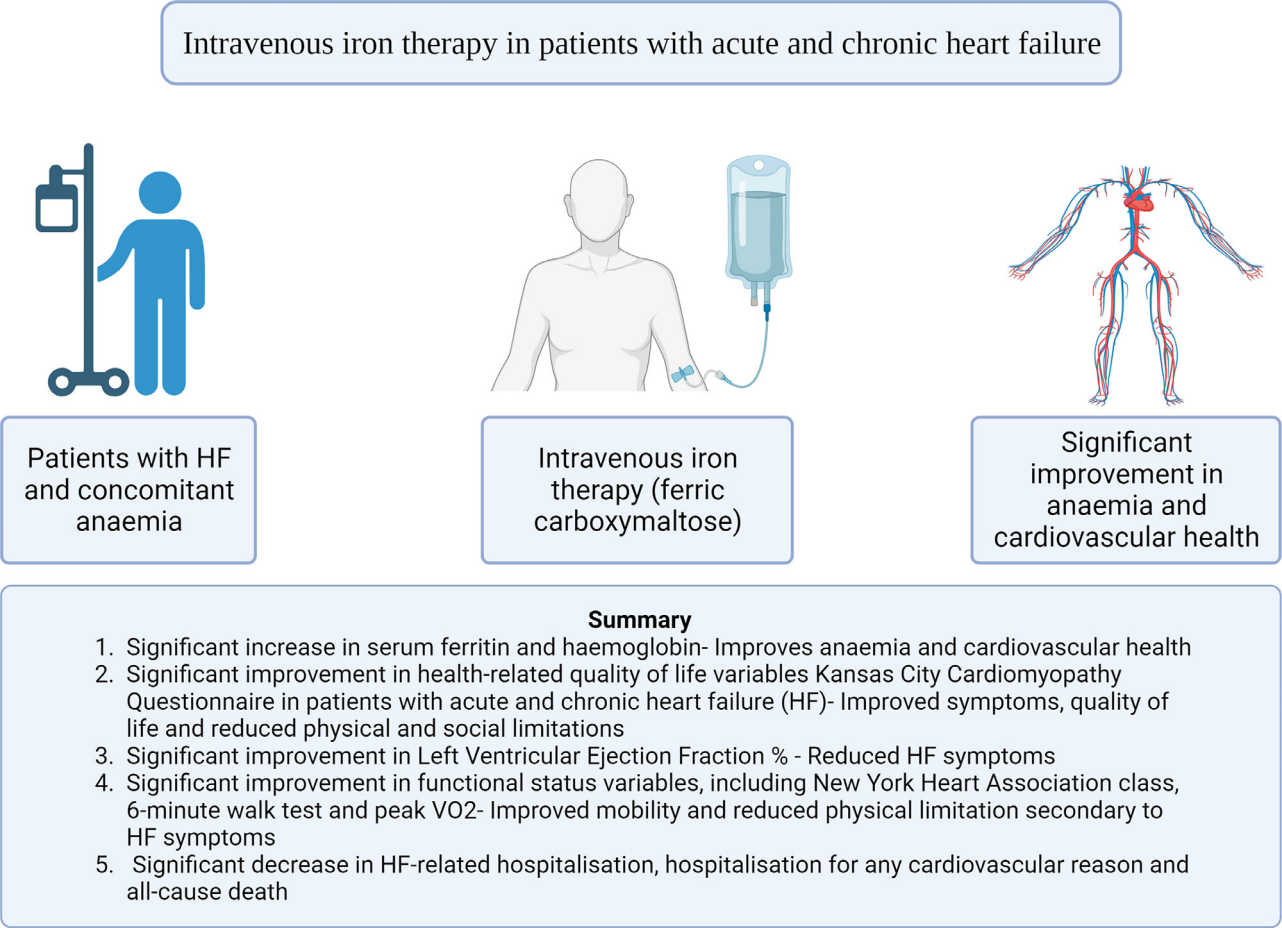
Summary of findings from the use of intravenous iron therapy in patients with heart failure and anaemia. This illustration was created using Biorender.com.

IV iron in patients with HFrEF showed statistically significance in improving clinical outcomes including an increase in haemoglobin, LVEF %, and serum ferritin in patients with HF. Additionally, a significant improvement in health-related quality of life (KCCQ), and functional status (NYHA, 6MWT, and peak VO_2_) variables in patients with acute and chronic HF. The improvement in LVEF % is of debateable clinical significance, and its effect on the patients’ clinical, HRQoL, and functional status outcomes is unclear. Moreover, despite the lack of clinical significance in all-cause hospitalisation and HF-related deaths, a significant decrease in HF hospitalisation, hospitalisation from any cardiovascular reason and all-cause death which supports the need for implementation of IV iron as a standard of care in patients HF and iron deficiency. A summary of our findings are displayed in Fig. 11.

## Declaration of competing interest

The authors declare that they have no known competing financial interests or personal relationships that could have appeared to influence the work reported in this paper.

## Appendix Supplementary materials

Image, application 1

Image, application 2

Image, application 3
