# Supplementary material for: Intravenous iron for acute and chronic heart failure with reduced ejection fraction (HFrEF) patients with iron deficiency: An updated systematic review and meta-analysis
Source: Clin Med (Lond). 2024 Apr 21;24(3):100211. doi: 10.1016/j.clinme.2024.100211 (PMC11092397; doi:10.1016/j.clinme.2024.100211)

Appendix 1

Supplementary figure 1: PRISMA flow diagram.

Supplementary figure 2: ROB-2 of our included studies

Supplementary figure 1: PRISMA flow diagram.

**Identification of studies via databases and registers**

Records identified from*:

Databases (n =937)

PubMed (n=344)

Cochrane (n=102)

WOS (n=316)

Scopus (n=385)

Records removed *before screening*:

Duplicate records removed (n = 42)

**Identification**

Records screened

(n = 895)

Records excluded**

(n =870)

Reports sought for retrieval

(n = 25)

Reports not retrieved

(n =0)

**Screening**

Reason reports excluded:

Irrelevant (n =3)

Reviews (n = 2)

Letter to editors (n =1)

Conference paper (n=1)

Reports assessed for eligibility

(n =25)

Studies included in review

(n =18)

Reports of included studies

(n =18)

**Included**

*From:*  Page MJ, McKenzie JE, Bossuyt PM, Boutron I, Hoffmann TC, Mulrow CD, et al. The PRISMA 2020 statement: an updated guideline for reporting systematic reviews. BMJ 2021;372:n71. doi: 10.1136/bmj.n71

For more information, visit: <http://www.prisma-statement.org/>

Supplementary figure 2: ROB-2 of our included studies


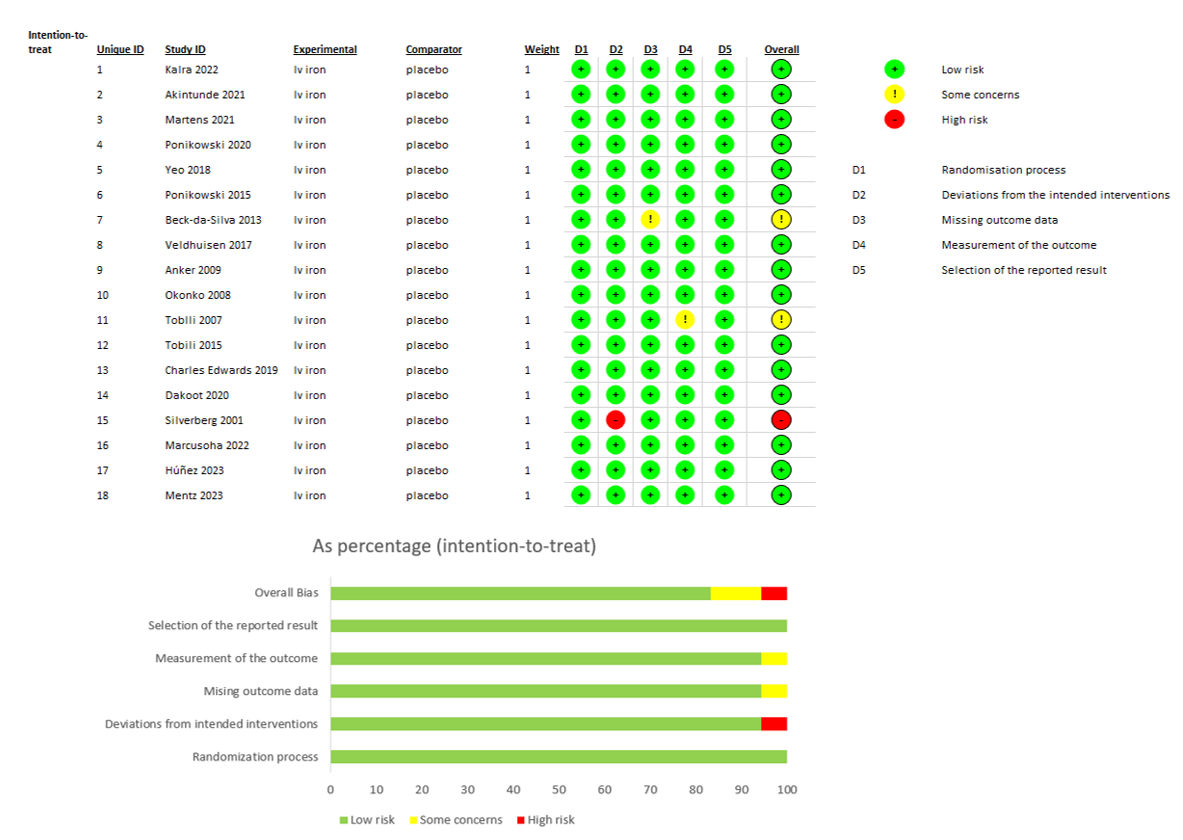

Supplement: Supplementary file 1 [file mmc1.docx]
