# Supplementary material for: Intravenous iron for acute and chronic heart failure with reduced ejection fraction (HFrEF) patients with iron deficiency: An updated systematic review and meta-analysis
Source: Clin Med (Lond). 2024 Apr 21;24(3):100211. doi: 10.1016/j.clinme.2024.100211 (PMC11092397; doi:10.1016/j.clinme.2024.100211)
Supplement: Supplementary file 2 [file mmc2.docx]

Appendix 2

Supplementary table 1: PRISMA checklist

Supplementary Table 2: Comprehensive overview of the included trials

Supplementary table 1: PRISMA checklist

| **Section and Topic** | **Item #** | **Checklist item** | **Location where item is reported** |
| --- | --- | --- | --- |
| **TITLE** | | |  |
| Title | 1 | Identify the report as a systematic review. | 1 |
| **ABSTRACT** | | |  |
| Abstract | 2 | See the PRISMA 2020 for Abstracts checklist. | 3 |
| **INTRODUCTION** | | |  |
| Rationale | 3 | Describe the rationale for the review in the context of existing knowledge. | 5 |
| Objectives | 4 | Provide an explicit statement of the objective(s) or question(s) the review addresses. | 5 |
| **METHODS** | | |  |
| Eligibility criteria | 5 | Specify the inclusion and exclusion criteria for the review and how studies were grouped for the syntheses. | 6 |
| Information sources | 6 | Specify all databases, registers, websites, organisations, reference lists and other sources searched or consulted to identify studies. Specify the date when each source was last searched or consulted. | 6 |
| Search strategy | 7 | Present the full search strategies for all databases, registers and websites, including any filters and limits used. | 6 |
| Selection process | 8 | Specify the methods used to decide whether a study met the inclusion criteria of the review, including how many reviewers screened each record and each report retrieved, whether they worked independently, and if applicable, details of automation tools used in the process. | 6 |
| Data collection process | 9 | Specify the methods used to collect data from reports, including how many reviewers collected data from each report, whether they worked independently, any processes for obtaining or confirming data from study investigators, and if applicable, details of automation tools used in the process. | 6 |
| Data items | 10a | List and define all outcomes for which data were sought. Specify whether all results that were compatible with each outcome domain in each study were sought (e.g. for all measures, time points, analyses), and if not, the methods used to decide which results to collect. | 7 |
|  | 10b | List and define all other variables for which data were sought (e.g. participant and intervention characteristics, funding sources). Describe any assumptions made about any missing or unclear information. | 7 |
| Study risk of bias assessment | 11 | Specify the methods used to assess risk of bias in the included studies, including details of the tool(s) used, how many reviewers assessed each study and whether they worked independently, and if applicable, details of automation tools used in the process. | 7 |
| Effect measures | 12 | Specify for each outcome the effect measure(s) (e.g. risk ratio, mean difference) used in the synthesis or presentation of results. | 7 |
| Synthesis methods | 13a | Describe the processes used to decide which studies were eligible for each synthesis (e.g. tabulating the study intervention characteristics and comparing against the planned groups for each synthesis (item #5)). | 7 |
|  | 13b | Describe any methods required to prepare the data for presentation or synthesis, such as handling of missing summary statistics, or data conversions. | 7 |
|  | 13c | Describe any methods used to tabulate or visually display results of individual studies and syntheses. | 7 |
|  | 13d | Describe any methods used to synthesize results and provide a rationale for the choice(s). If meta-analysis was performed, describe the model(s), method(s) to identify the presence and extent of statistical heterogeneity, and software package(s) used. | 7 |
|  | 13e | Describe any methods used to explore possible causes of heterogeneity among study results (e.g. subgroup analysis, meta-regression). | 7 |
|  | 13f | Describe any sensitivity analyses conducted to assess robustness of the synthesized results. | 7 |
| Reporting bias assessment | 14 | Describe any methods used to assess risk of bias due to missing results in a synthesis (arising from reporting biases). | 7 |
| Certainty assessment | 15 | Describe any methods used to assess certainty (or confidence) in the body of evidence for an outcome. | 7 |
| **RESULTS** | | |  |
| Study selection | 16a | Describe the results of the search and selection process, from the number of records identified in the search to the number of studies included in the review, ideally using a flow diagram. | 8 |
|  | 16b | Cite studies that might appear to meet the inclusion criteria, but which were excluded, and explain why they were excluded. | 8 |
| Study characteristics | 17 | Cite each included study and present its characteristics. | 8 |
| Risk of bias in studies | 18 | Present assessments of risk of bias for each included study. | 8 |
| Results of individual studies | 19 | For all outcomes, present, for each study: (a) summary statistics for each group (where appropriate) and (b) an effect estimate and its precision (e.g. confidence/credible interval), ideally using structured tables or plots. | 8 |
| Results of syntheses | 20a | For each synthesis, briefly summarise the characteristics and risk of bias among contributing studies. | 9, 10, 11 |
|  | 20b | Present results of all statistical syntheses conducted. If meta-analysis was done, present for each the summary estimate and its precision (e.g. confidence/credible interval) and measures of statistical heterogeneity. If comparing groups, describe the direction of the effect. | 9, 10, 11 |
|  | 20c | Present results of all investigations of possible causes of heterogeneity among study results. | 9, 10, 11 |
|  | 20d | Present results of all sensitivity analyses conducted to assess the robustness of the synthesized results. | 9, 10, 11 |
| Reporting biases | 21 | Present assessments of risk of bias due to missing results (arising from reporting biases) for each synthesis assessed. | 8 |
| Certainty of evidence | 22 | Present assessments of certainty (or confidence) in the body of evidence for each outcome assessed. | 8 |
| **DISCUSSION** | | |  |
| Discussion | 23a | Provide a general interpretation of the results in the context of other evidence. | 12 |
|  | 23b | Discuss any limitations of the evidence included in the review. | 12 |
|  | 23c | Discuss any limitations of the review processes used. | 13 |
|  | 23d | Discuss implications of the results for practice, policy, and future research. | 13 |
| **OTHER INFORMATION** | | |  |
| Registration and protocol | 24a | Provide registration information for the review, including register name and registration number, or state that the review was not registered. | 6 |
|  | 24b | Indicate where the review protocol can be accessed, or state that a protocol was not prepared. | 6 |
|  | 24c | Describe and explain any amendments to information provided at registration or in the protocol. | 6 |
| Support | 25 | Describe sources of financial or non-financial support for the review, and the role of the funders or sponsors in the review. | 16 |
| Competing interests | 26 | Declare any competing interests of review authors. | 16 |
| Availability of data, code and other materials | 27 | Report which of the following are publicly available and where they can be found: template data collection forms; data extracted from included studies; data used for all analyses; analytic code; any other materials used in the review. | 16 |

*From:*  Page MJ, McKenzie JE, Bossuyt PM, Boutron I, Hoffmann TC, Mulrow CD, et al. The PRISMA 2020 statement: an updated guideline for reporting systematic reviews. BMJ 2021;372:n71. doi: 10.1136/bmj.n71

For more information, visit: <http://www.prisma-statement.org/>

Supplementary Table 2: comprehensive overview of the included trials

| Study ID | Study design, country | Sample size of each group | Criteria | study duration | Iron treatment regimen | Conclusion |
| --- | --- | --- | --- | --- | --- | --- |
| Kalra 2022 | RCT,  United Kingdom | Ferric derisomaltose  group (n=569)  Usual care group  (n=568) | - Age 18 years or older - LVEF ≤45% based on the most recent assessment within the past two years using any conventional imaging method - NYHA class II - IV, indicating moderate to severe heart failure symptoms - Iron deficiency defined as TSAT <20% and/or ferritin <100 ug/L - Higher risk HF group, demonstrated by either current or recent hospitalization for HF (within 6 months) or out-patients with elevated NT-proBNP levels (>250 ng/L in sinus rhythm or >1,000 ng/L in atrial fibrillation), or elevated BNP levels (>75 pg/mL or 300 pg/mL, respectively) - Must be able and willing to provide informed consent. | 20 months | - Patients <50 kg: 20 mg/kg - Patients 50-<70 kg: 1000 mg if Hb ≥10 g/dL, or 20 mg/kg if Hb <10 g/dL - Patients ≥70 kg: 20 mg/kg (up to 1500 mg if Hb ≥10 g/dL, or 2000 mg if Hb <10 g/dL) | For a broad range of patients with heart failure, reduced left ventricular ejection fraction and iron  deficiency, intravenous ferric derisomaltose administration was associated with a lower risk of hospital admissions  for heart failure and cardiovascular death, further supporting the benefit of iron repletion in this population. |
| Akintunde 2021 | Retrospective cohort,  Nigeria | Iron replacement  group (n=30)  Controls (n=30) | Participants included in the study:   - Followed up for heart failure in the clinic for ≥6 months - Adults aged >18 years Provided informed consent   Data collection:   - Used a form to record clinical, laboratory, and demographic parameters Heart failure diagnosis: - Based on recent European Society of Cardiology guidelines.. | 2 months | Participants received an average of 1,000 g of parenteral iron dextran infusion over 3-5 weeks, divided into multiple doses. | Parenteral iron dextran therapy was well tolerated in heart failure patients and associated with improved quality of life, clinical profile, and functional classification in Nigerian individuals with heart failure. Iron replacement therapy can be a beneficial treatment option for African patients with heart failure to enhance prognosis. |
| Martens 2021 | RCT,  Belgium | Standard of care (n=538) Ferric carboxylates (n=537) | - Chronic heart failure with cardiac resynchronization therapy implanted over 6 months ago - Presence of iron deficiency (ferritin < 100 μg/l, regardless of TSAT, or ferritin between 100-300 μg/l with TSAT < 20%) - Presence of incomplete reverse remodeling (LVEF < 40%) - Age ≥18 years Informed consent obtained Stable pharmacological therapy for heart failure in the past 4 weeks (excluding diuretics) | 3months | - Active treatment: Intravenous iron administration using FCM (InjectaferVR/Ferinject VR) diluted in 250mL NaCl 0.9%. - Placebo intervention: 250mL NaCl 0.9% without FCM. - Dosing scheme: Patients required a dose of FCM ranging between 500 and 2000mg. - Maximum allowed dose: 1000mg of FCM per week. - Follow-up appointment: Patients needing a dose of 1500 or 2000mg received a follow-up appointment after 1-2 weeks to receive the remaining dose. | Treatment with FCM (Ferric Carboxymaltose) in HFrEF (Heart Failure with Reduced Ejection Fraction) patients who have iron deficiency and persistently reduced LVEF after CRT (Cardiac Resynchronization Therapy) leads to improvement in cardiac function. This improvement is measured by increased LVEF (Left Ventricular Ejection Fraction), decreased LVESV (Left Ventricular End-Systolic Volume), and enhancement of the cardiac force-frequency relationship. |
| Ponikowski 2020 | RCT, Argentina, Brazil, Croatia, Georgia, Israel, Italy, Lebanon, Netherlands, Poland, Romania, Singapore, Spain, Sweden, Ukraine, United Kingdom | Ferric  carboxymaltose  (n=558)  Placebo  (n=550) | - Persistent dyspnea at rest or with minimal exertion upon admission - Presence of at least 2 of the following clinical findings: congestion on chest X-ray, rales on chest auscultation, ≥1+ edema, elevated jugular venous pressure - Natriuretic peptide levels meeting specific thresholds based on heart rhythm and medication history - AHF episode treated with a minimum of 40 mg IV furosemide (or equivalent IV loop diuretic) - Iron deficiency defined as serum ferritin <100 ng/mL or 100 ng/mL ≤ serum ferritin ≤299 ng/mL if TSAT <20% - Left ventricular ejection fraction <50% assessed within 12 months prior to randomization - Male or female aged ≥18 years old - Written informed consent provided by the subject or legally acceptable representative. | 6 months | FCM (Ferric carboxymaltose) is given as a bolus IV injection. The dose depends on the participant's body weight and Hb level, with 10 ml or 20 ml of undiluted solution administered (containing 500 mg or 1,000 mg of iron respectively | In patients with iron deficiency, a left ventricular ejection fraction of less than 50%, and who were  stabilised after an episode of acute heart failure, treatment with ferric carboxymaltose was safe and reduced the risk  of heart failure hospitalisations, with no apparent effect on the risk of cardiovascular death. . |
| Yeo 2018 | RCT, Singapore. | FCM  (n = 24)  Saline  (n = 25) | - Clinical diagnosis of acute decompensated heart failure (based on European Society of Cardiology guidelines), regardless of left ventricular ejection fraction. - Iron deficiency defined as serum ferritin <300 ng/mL if transferrin saturation is <20%. - Able to complete the 6-min walk test. - Age 21 years and above. | 3months | Study drug: i.v. FCM solution (Ferinject®, Vifor Pharma). Dose: Single dose of 1000 mg (50 mg of iron per milliliter). Administration: Undiluted i.v. bolus injection of 20 mL over 15 minutes. Placebo: Equivalent volume (20 mL) of i.v. 0.9% saline. | Intravenous FCM administered pre-discharge in Southeast Asians hospitalized with decompensated HF is  clinically feasible. Changes in 6MWT distance should be measured beyond Week 12 to account for background therapy  effects. . |
| Ponikowski 2015 | RCT,Poland, Russian Federation | FCM (N= 150) Placebo(N=151) | - ron deficient subjects with stable chronic heart failure (CHF) (NYHA II-III) on optimal background therapy for CHF - Reduced left ventricular ejection fraction - Capable of completing 6 minute walk test - At least 18 years of age and with written informed consent prior to any study specific procedures | 52 months | Intravenous iron: Ferric carboxymaltose solution (FCM) [Ferinject/Injectaferw Vifor Pharma].  Study medication: Undiluted bolus i.v. injections of 10 or 20 mL. 10 mL: Equivalent to 500 mg of iron. 20 mL: Equivalent to 1000 mg of iron.  Administration: Administered over at least 1 minute.  Placebo: Normal saline [0.9% weight/volume (w/v) NaCl] administered according to active therapy instructions. | Treatment of symptomatic, iron-deficient HF patients with FCM over a 1-year period resulted in sustainable improvement  in functional capacity, symptoms, and QoL and may be associated with risk reduction of hospitalization for worsening  HF |
| Beck-da-Silva 2013 | RCT, Brazil | Group 1 : Iron Sucrose N=10 Group 2 : Ferrous sulfate N=7  Group 3 : Placebo N=6 | - Age 18 years or older. - Outpatients followed at a HF clinic in a tertiary care hospital. - Clinical diagnosis of HF for at least 3 months before study entry. - NYHA functional class II-IV. - Able to perform spirometry. - Documentation of LVEF <40% within the last 6 months. - Adequate baseline therapy for HF based on patient's functional class (β-blockers, ACE inhibitors, digoxin, spironolactone if NYHA class III or IV). - Stable baseline HF therapy with no intent to increase doses for the following 3 months. - Hemoglobin ≤12 g/dl and ≥9 g/dl. - Transferrin saturation <20% and ferritin <500 μg/l. - Ability to provide written informed consent. | 3-month | Group 1: Iron Sucrose 200 mg intravenously, once a week, in 30 min infusions, for 5 weeks and placebo of oral presentation, three times a day, for 8 weeks.  Group 2: Ferrous sulphate 200 mg, orally, three times a day, for 8 weeks and placebo of IV presentation once a week, for 5 weeks.  Group 3: Placebo of oral presentation, three times a day, for 8 weeks and placebo of IV presentation once a week, for 5 weeks. | There was no statistically significant change in oxygen consumption in the PO iron group. Whereas, ferritin level and Transferrin Saturation were significantly  increased in both intervention groups. However, the correction of haemoglobin level seems to be similar in all the groups. Thus, IV iron shows better results compared to PO iron in improving functional capacity in patients with heart failure. |
| Veldhuisen 2017 | RCT, Australia, Belgium , The Netherlands, Poland, Italy, Germany, Russia, United Kingdom, Switzerland, France, Spain | Iron replacement  group (n=86)  PLACEBO (n=86) | - Age ≥18 years. - Clinically stable mild to moderate chronic HF (NYHA functional class II-III). - On optimal background therapy for HF for ≥4 weeks with no recent dose changes. - Left ventricular ejection fraction ≤45% assessed within ≤3 months of screening and >3 months after stable β-blocker therapy or device implantation. - Baseline plasma brain natriuretic peptide (BNP) concentration >100 pg/mL or N-terminal (NT) proBNP >400 pg/mL. - Decreased exercise capacity with reproducible peak VO2 of 10 to 20 mL/kg/min. - Documented iron deficiency: serum ferritin <100 ng/mL or serum ferritin of 100 to 300 ng/mL with transferrin saturation (TSAT) <20%. | 6 months | intravenous iron administration:  Undiluted intravenous bolus injection given in ≥1 minute.  Infusion of 10 or 20 mL of ferric carboxymaltose (FCM) diluted in ≈100 mL or ≈200 mL of sterile 0.9% sodium chloride solution, respectively.  Infusion time of ≥6 minutes for 10 mL and ≥15 minutes for 20 ml | Treatment with intravenous FCM showed an improvment in iron storage and had a significant effect on peak VO2 was observed on FCM, compared with standard of care . |
| Anker 2009 | RCT, Switzerland, Russia, Ukraine, Germany | Iron replacement  group (n=304)  PLACEBO (n=155) | - Ambulatory patients with chronic heart failure of NYHA class II or III. - Left ventricular ejection fraction of 40% or less (for NYHA class II) or 45% or less (for NYHA class III) documented. - Hemoglobin level between 95 and 135 g/L at the screening visit. - Presence of iron deficiency. | 6 months | Administration of ferric carboxymaltose or saline:  Intravenous bolus injection of 4 mL.  Weekly dosing until iron repletion was achieved.  Maintenance phase started at week 8 or week 12, depending on the required iron-repletion dose.  During the maintenance phase, dosing was done every 4 weeks. | Significant improvements were seen in the intervention group who were receiving intravenous ferric carboxymaltose in terms of symptoms, functional capacity, the 6-minute walk test, and quality of life compared to placebo group , these improvements concern patients with chronic heart failure and iron deficiency, with or without anemia. The number of death and adverse events were similar in the two study groups. |
| Okonko 2008 | RCT, UK, Poland | Iron replacement  group (n=24)  PLACEBO (n=11) | - Age 21 years or older. - Symptomatic chronic heart failure (New York Heart Association [NYHA] functional class II or III). - Exercise limitation with a reproducible peak oxygen uptake (pVO2/kg) of 18 ml/kg/min during screening. - Average of two screening haemoglobin (Hb) concentrations: 12.5 g/dl for the anaemic group or 12.5 to 14.5 g/dl for the non-anaemic group. - Ferritin level of 100 g/l or between 100 g/l and 300 g/l with a transferrin saturation (TSAT) of at least 20%. - Left ventricular ejection fraction of 45% measured within the past 6 months using echocardiography or magnetic resonance imaging. - Use of maximally tolerated doses of optimal chronic heart failure therapy for at least 4 weeks before recruitment, with no dose changes for at least 2 weeks. - Resting blood pressure of 160/100 mm Hg or lower. - Normal red cell folate and vitamin B12 levels. | 4 months | Iron sucrose was provided as a solution for IV infusion in 5-ml ampules (20 mg iron/ml). The treatment group received iron weekly (therapeutic phase) then at weeks 4, 8, 12 and 16 (maintenance phase). | Treatment with Intravenous iron seems to effective in the improvements of exercise capacity and symptoms in patients with CHF . |
| Toblli 2007 | Retrospective cohort, Argentina | Iron replacement  group (n=20)  PLACEBO (n=20) | - Left ventricular ejection fraction (EF) ≤35% - New York Heart Association (NYHA) functional class II to IV - Anemia with an iron deficit defined by hemoglobin (Hb) ≤12.5 g/dl for men and ≤11.5 g/dl for women, and with additional criteria of serum ferritin ≤100 ng/ml and/or transferrin saturation (TSAT) ≤20% - Creatinine clearance ≥90 ml/min. | 6 months | IV ISC 200 mg weekly for 5 weeks. | Treatment with intravenous iron was associated with significant improvements compared to placebo group with regards to NT-proBNP level , inflammatory status, LVEF, NYHA functional class, exercise capacity, renal function, and better quality of life. |
| Toblli 2015 | Retrospective cohort, Argentina | Iron replacement  group (n=30)  PLACEBO (n=30) | - LVEF of 35%, - NYHA functional class II to IV, Hb <12.5 g/dL (men) or Hb <11.5 g/dl (women), - ferritin <100 ng/ml and/or percentage transferrin saturation (TSAT) 20%, and creatinine clearance (CrCl) 90 mL/min. | 6 months | he intervention group (n=30) received 200 mg/200 mL of iv IS every week for five weeks in addition to conventional therapy | Intravenous iron treatment was associated with improved myocardial functional parameters and cardiac dimensions in patients with anaemia and chronic kidney disease. |
| Charles Edwards 2019 | Retrospective cohort, United Kingdom | Iron replacement  group (n=21)  PLACEBO (n=19) | - Age ≥30 years - Stable symptomatic chronic heart failure (New York Heart Association [NYHA] class III) and left ventricular ejection fraction (LVEF) ≤45%, or if NYHA class II, LVEF ≤40% within the preceding 6 months - Use of optimal heart failure drugs for ≥4 weeks without dose changes for ≥2 weeks - Screening haemoglobin (Hb) <120 g/L in women and <130 g/L in men (anaemic group), or ≥120 g/L in women and ≥130 g/L in men (nonanemic group) - Iron deficiency (ID) defined by ferritin <100 μg/L or 100 to 300 μg/L with transferrin saturation <20% - Folate and vitamin B12 levels ≥lower limit of reference range - Resting blood pressure ≤160/100 mmHg - Negative pregnancy test in women of childbearing age. | 2 weeks | Iron isomaltoside was provided as a solution for intravenous infusion in 100 mg iron/mL ampoules. Monofer was added to 100 mL of sterile 0.9% saline for infusions. | In patients with chronic HF and iron deficiency, a total repletion dose of iron isomaltoside given at a single sitting is well tolerated and associated with faster skeletal muscle PCr t1/2 at 2 weeks. |
| Dahoot 2020 | Retrospective cohort United Kingdom | Iron replacement  group (n=35)  PLACEBO (n=35) | - symptomatic with chronic heart failure (NYHA Functional Class II/III) of age ≥18–65 years with iron deficiency, | 3months | IV FCM solution was administered to eligible subjects in 0.9% normal saline bolus over 1 h | The outcome showed significant benefit in symptoms and improvement in quality of life. Therefore, simple intravenous iron replacement along with other heart failure measures can make life easier for patients with heart failure. |
| Silverberg, 2001 | Retrospective cohort, Israel | Iron replacement  group (n=16)  PLACEBO(n=16) | - ¨Patients with severe CHF (NYHA class $III), with fatigue and/or shortness of breath on even mild exertion or at rest, - levels of Hb in the range of 10 to 11.5 g% on at least three consecutive visits over a three-week period, - LVEF of ,40%. | range 5 to 12 months | All patients in Group A received the combination of sc EPO and IV Fe. The EPO was given once a week at a starting dose of 4,000 international units (IU) per week sc and the dose was increased to two or three times a week or decreased to once every few weeks as necessary. The IV Fe (Venofer-Vifor International, Switzerland), a ferric sucrose product, was given in a dose of 200 mg IV in 150 ml saline over 60 min every two weeks | In patients treated with IV iron, a marked improvement in cardiac and patient function is seen, associated with less hospitalization and renal impairment and less need for diuretics. |
| Marcusohn 2022 | RCT, Israel | Iron replacement  group (n=18)  PLACEBO(n=16) | - haemoglobin levels of 8–14 mg/dL on admission, - ferritin levels ,100 ng/mL or ferritin 100–300 ng/Ml with transferrin saturation ,20%, - N-terminal pro–Btype natriuretic peptide (NT-proBNP) level 300 pg/mL (or 800 pg/mL in the presence of atrial fibrillation), and - treatment with IV loop diuretics | 6 months | IV SGFC 125 mg | IV SFGC–treated patients had a comparable 6-minute  walk at 3 and 6 months despite suffering from more severe HF with  higher baseline NT-proBNP |
| Núñez 2020 | RCT, Spain | Iron replacement  group (n=27)  PLACEBO(n=26) | - ▪ Outpatients with chronic HF - ▪ Oder than 18 years - ▪ NYHA II-III with optimal medical treatment in the last 4 weeks, without dose - changes of HF treatment in the last 2 weeks (except for diuretics) - ▪ NT-proBNP >400 pg/mL - ▪ LVEF <50% in the last 12 months - ▪ ID, defined as: serum ferritin <100μg/L, or 100-299μg/L if TSAT <20% and - Haemoglobin <15 g/dL - ▪ Participants are willing and able to give informed consent for participation in the - study | 1 months | FCM solution was given as a 20-mL perfusion (equivalent to  1000 mg of iron) diluted in a sterile saline solution (0.9% wt/  vol NaCl) | —In patients with heart failure and iron deficiency, FCM administration was associated with changes in the T2* and T1  mapping cardiac magnetic resonance sequences, indicative of myocardial iron repletion |
| Mentz 2023 | RCT, USA | Iron replacement  group (n=1532)  PLACEBO(n=1533) | - Eligible patients: Adults (≥18 years) with heart failure - Left ventricular ejection fraction: 40% or less - Haemoglobin level: Greater than 9.0 g/dL - Haemoglobin criteria for women: Less than 13.5 g/dL - Haemoglobin criteria for men: Less than 15.0 g/dL - Iron deficiency criteria: Ferritin <100 ng/mL or 100-300 ng/mL with transferrin saturation <20% - Additional criteria: Either hospitalized for heart failure in the past 12 months or elevated natriuretic peptide level | 12 months | Day 0 and Day 7:  Group A (FCM): 750 mg undiluted IV FCM at ~100 mg (2 mL)/min  Group B (Placebo): Blinded placebo (15 cc normal saline) IV push at 2 mL/min | There was no significant difference between ferric carboxymaltose and placebo among patients with heart failure with a reduced ejection fraction and iron deficiency |
